# Supplementary material for: Conditional chemoconnectomics (cCCTomics) as a strategy for efficient and conditional targeting of chemical transmission
Source: eLife. 2024 Apr 30;12:RP91927. doi: 10.7554/eLife.91927 (PMC11060718; doi:10.7554/eLife.91927)
Supplement: Figure 3—source data 3. [file elife-91927-fig3-data3.zip › Figure 3 source data3 part 1.zip!/Figure 3-Source data 3-part1/1067-9-CRISPResso_Report_c33kf3u3.zip!/CRISPResso_on_1067_9/3a.Indel_size_distribution.pdf]

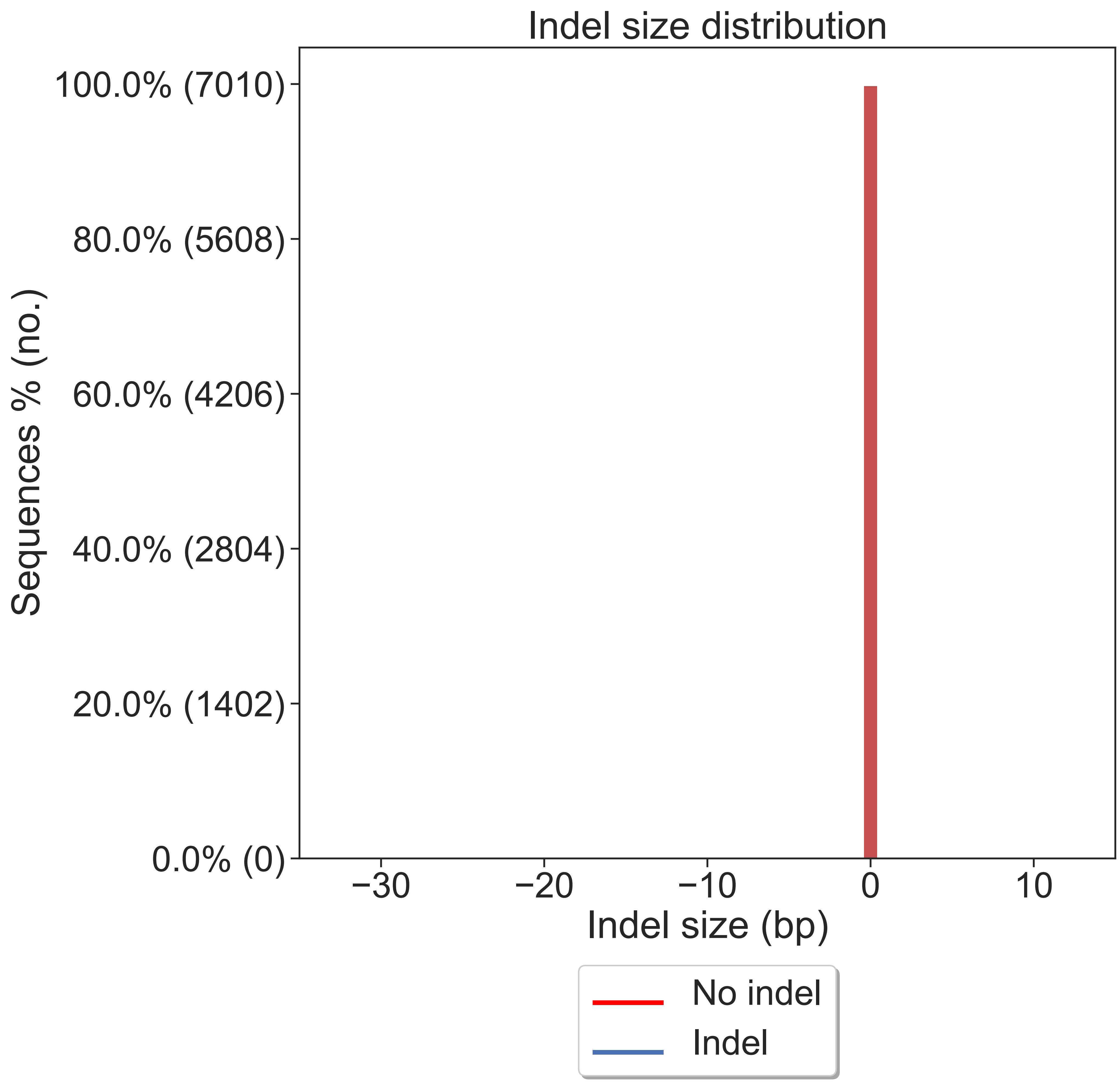
Indel size distribution
100.0% (7010)
80.0% (5608)
60.0% (4206)
40.0% (2804)
20.0% (1402)
0.0% (0)
Sequences % (no.)
−30
−20
−10
0
10
Indel size (bp)
No indel
Indel
